# Biomechanics of oral mucosa

**DOI:** 10.1098/rsif.2015.0325

**Published:** 2015-08-06

**Authors:** Junning Chen, Rohana Ahmad, Wei Li, Michael Swain, Qing Li

**Affiliations:** 1 School of Aerospace, Mechanical and Mechatronic Engineering, University of Sydney, Sydney, New South Wales 2006, Australia; 2 Faculty of Dentistry, University of Sydney, Sydney, New South Wales 2006, Australia; 3 Unit of Prosthodontics, Faculty of Dentistry, Universiti Teknologi MARA, Shah Alam 40450, Malaysia

**Keywords:** oral mucosa, hyperelastic, viscoelastic, hydrostatic pressure, pressure–pain threshold, residual ridge resorption

## Abstract

The prevalence of prosthodontic treatment has been well recognized, and the need is continuously increasing with the ageing population. While the oral mucosa plays a critical role in the treatment outcome, the associated biomechanics is not yet fully understood. Using the literature available, this paper provides a critical review on four aspects of mucosal biomechanics, including static, dynamic, volumetric and interactive responses, which are interpreted by its elasticity, viscosity/permeability, apparent Poisson's ratio and friction coefficient, respectively. Both empirical studies and numerical models are analysed and compared to gain anatomical and physiological insights. Furthermore, the clinical applications of such biomechanical knowledge on the mucosa are explored to address some critical concerns, including stimuli for tissue remodelling (interstitial hydrostatic pressure), pressure–pain thresholds, tissue displaceability and residual bone resorption. Through this review, the state of the art in mucosal biomechanics and their clinical implications are discussed for future research interests, including clinical applications, computational modelling, design optimization and prosthetic fabrication.

## 1. Introduction

With an increasing elderly population worldwide, the edentulous group of patients has been continuously expanding, resulting in significantly raised needs for prosthodontic treatments [1,2]. Over the past century, complete or partial dentures have been widely used in dental clinics to restore oral function [3–5]. During mastication, the oral mucosa beneath the denture plays a critical role in distributing occlusal loads to the underlying bony ridge over a large denture-supporting tissue interface [6–9]. Within this highly vascular tissue, the functional pressure, namely interstitial fluid pressure (IFP) or hydrostatic pressure, has been identified as one of the most important aetiological factors causing the accompanying clinical complications [9–14].

The mandible of the ageing patient is mainly supported by the periosteal plexus of blood vessels, and therefore is very susceptible to diminished circulation under occlusal load -induced mucosal pressure [15], which triggers nerve pain [16] and discomfort [14,17], thus compromising patients' life quality [18,19]. Cellular swelling, increased nuclear size, and intercellular oedema will occur when the mucosa is under compression [9,13,20]. The inflammatory response of cells and surrounding tissue further contributes to variation in permeability of the mucosal tissue and continues to compromise circulation [21,22]. Once the hydrostatic pressure builds up and exceeds the capillary pressure, blood flow will be decreased and may even temporarily cease altogether as a result of the combination of active arteriolar closure and passive capillary compression [22]. Consequently, reduced nutrient supply and metabolite removal may lead to residual ridge resorption [3,9,11,12,23–26], a progressive phenomenon harmful to patients' oral health [27,28].

It is critical to understand the mucosal response to prosthodontic prostheses for the treatment outcome, and the mucosa has been found to exhibit complex nonlinear and time-dependent behaviours since the investigations commenced more than five decades ago [29–33]. Significant interest has arisen and extensive studies have been conducted to explore the biomechanics of the mucosa both clinically and theoretically.

This paper aims to provide a systematic review of the biomechanics of mucosal responses to mechanical loading, and it is structured into three parts. Firstly, a brief summary of the mucosa anatomy and physiology will introduce the basic biology associated with its biomechanical responses and illustrate the insights associated with these observations. Secondly, a critical review is conducted of both experimental and numerical studies on four major aspects of the mucosal responses, namely static, dynamic, volumetric and interactive responses. Several material models for each individual aspect are investigated and three-dimensional finite-element models of the mucosa are compared. Finally, the clinical implications of mucosa biomechanics are discussed considering the major relevance to prosthodontic treatments, including the tissue remodelling stimulus, pressure–pain threshold (PPT), tissue displaceability and residual ridge resorption.

Understanding and adopting appropriate material models for the corresponding biomechanical behaviours will help identify biological determinants influencing the mucosa responses for planning and prediction of better prosthodontic treatment. Furthermore, this review will showcase the state of the art in mucosal biomechanics research and reveals the potential research opportunities on fundamental biomechanics, clinical applications and design optimization.

## 2. Anatomical and physiological factors

**Figure 1.**
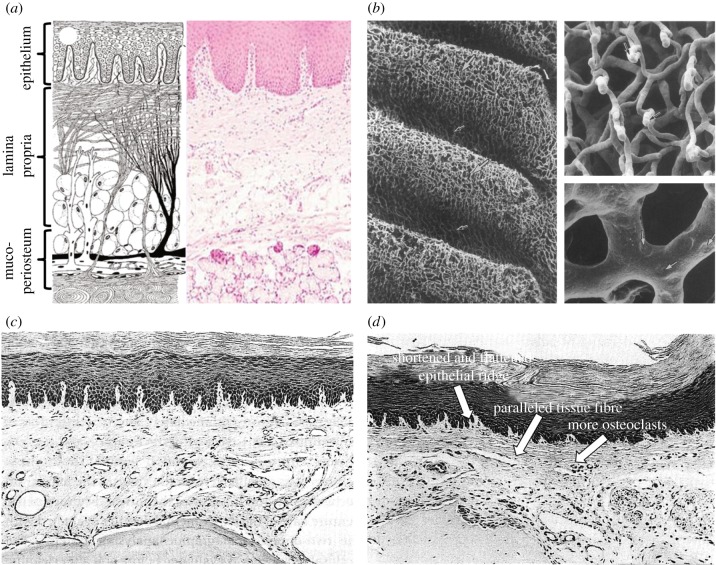
(*a*) Schematic diagram (left) and histological diagram of the healthy mucosal anatomy [34]; (*b*) SEM images of the vascular network within the rabbit palatine mucosa by corrosion casts [35]; (*c*) histological image of the mouse mucosa underneath the denture without occlusal load [13] and (*d*) histological image of the mouse mucosa beneath a denture [13].

The masticatory mucosa exhibits distinct resistance to deformation under load [21], which comprises a surface epithelial layer and a deeper connective tissue layer, namely the lamina propria (figure 1*a*). The former consists of multiple rows of cells that constitute a load-bearing layer by intercellular adhesions. Within this layer, intercellular channels exist for communication with neighbouring cells and contain viscous material (mucopolysaccharides) providing deformability and bearing load [21,36]. The underlying lamina propria is a compact fibrous tissue, comprising two sub-layers, the papillary layer and the deeper reticular layer. The superficial collagen fibres in the papillary layer are randomly oriented, and the transient regions to the epithelium are often irregular and non-smooth with undulating papillae ridges, providing enlarged areas for nutrient transport [21]. The basal collagen fibres in the reticular layer gradually orient to perpendicularly attach the periosteum. The abundance of such fibrous attachments, known as mucoperiosteum, renders the oral mucosa immovable with firm connection to the bone, resisting compression and shear in function [34,37]. The entire mucosa thickness can vary over a wide range [6,38–45], from 0.30 mm on the attached buccal mucosa in the canine mandible to 6.7 mm in the maxillary tuberosity region. It has been determined as one of the dominant factors to affect their biomechanical responses [8], aside from its various types and locations [29,30,35].

Along with the anatomical features, the oral mucosa also plays a critical physiologic role in distributing masticatory forces, protecting the underlying residual ridge from excessive loading [6–9]. As a highly vascularized tissue (figure 1*b*), the mucosa contains a considerable amount of interstitial fluid, and its protective function arises from the mechanical cushioning effect [46]. The pressure induced by dentures provides a pumping effect for pushing the interstitial fluid to the unloaded neighbouring tissues [21,32]. With the movement of fluid, the collagen fibres are forced to align along the lines of mechanical stresses, passively protecting the connective tissue itself and underlying bone. With increasing masticatory loads, the IFP builds up [9,11,12]. Once IFP exceeds the vascular pressure, blood flow will be reduced and may temporarily cease, potentially leading to localized ischaemia [22,26,47–49]. This is a time-dependent process and increases with loading time until a plateau. The degree of ischaemia depends on the loading magnitude and duration. The prolonged interference of blood flow further induces local anoxia and accumulation of metabolites, leading to the destruction of the supporting bony tissues, known as residual ridge resorption [9,10,13,47,50].

Upon mechanical load release, the mucosa is capable of recovery at different extents [7,29,30,51], and the released surface pressure allows the interstitial fluid to flow back [52,53]. While the load-induced impedance of blood supply is not an irreversible condition, the recovery time is proportional to the loading magnitude and duration but the extent of recovery is converse [26,47,49,54]. In young subjects, the blood flow can be almost fully restored following a short loading, and the recovery may even exceed the initial blood flow by as much as 10% [26]. Therefore, the intermittent masticatory pressure may even improve circulation. By contrast, more permanent effects of lowering blood supply may result from wearing dentures for over six months [49]. Ischaemia occurs with continuous clenching and delays the recovery of blood flow in the mucosa after release of compression. Continuous pressure over a prolonged duration may even alter the oral anatomy, consequently affecting the physiological responses [51]. Minimal histological changes were found with narrowed epithelial ridges without an occlusal load [55] (figure 1*c*), while an active load induced inflammatory change and alveolar bone remodelling [9,13], followed by severely reduced epithelium thickness (exceeding 30%) [48], manifesting the shortened and branching epithelial ridges [9,13] (figure 1*d*). The mucosa then becomes less resilient to masticatory forces and more sensitive to pressure [56].

The mucosa exhibits a higher tolerance to intermittent than continuous pressure, as the threshold for the alveolar ridge resorption was 19.6 kPa for the former and 6.86 kPa for the latter [10]. A high level of continuous pressure can induce more severe ridge resorption [11]. At the other extreme, continuous pressure less than 1.96 kPa (9.8 kPa for intermittent) caused no bone resorption [10], though apposition was also inhibited [11]. Clinical recommendation was made based on these findings, that the patients should remove their dentures during sleep to aid recovery of blood supply to the palatal mucosa [57]. In the patients with chronic diseases or conditions, e.g. diabetes mellitus or osteoporosis, the oral mucosa and underlying bone are more sensitive to occlusal loads, as shown by the lowered thresholds [58–61].

## 3. Biomechanical responses

While there are many aspects of the biomechanical responses of the oral mucosa, this study will focus on the four key biomechanical issues that are closely relevant to clinical applications, thereby revealing the biological insights to these mechanical models. The first one is the static response, which is often known as the short-term or instant response. It is often modelled as the elasticity of a material in a path-dependent manner. The second one is the dynamic response, or the so-called long-term and delayed response. It can be induced by the viscosity or permeability of the fluid component in the soft tissue, and interpreted in a time-dependent process. The third is the volumetric response, determined by the compressibility or Poisson's ratio to indicate the capability of resisting a volumetric change while the shape is deformed. The last one is the surface interactive response, which is represented by the friction coefficients between the mucosa and prosthetic materials.

### 3.1. Elasticity

As one of the fundamental parameters to define material behaviour, the modulus of elasticity is the physical description of an object's tendency to be deformed proportionally to the applied force. The oral mucosa was found to be highly deformable under compression [62], and the elastic modulus appears to vary over a broad range. Being a heterogeneous material, the mucosal instant stiffness results from both the solid matrix structure (e.g. epithelial layer, fibrous network, blood vessel, etc.) and the fluid components (e.g. interstitial fluid, blood). Several material models have been developed to interpret such mucosal behaviours, including linear elastic, biphasic, multi-phasic elastic and hyperelastic models. Within a short instant loading, the mass transfer, such as the fluid flow, is often disregarded in these models. In other words, this aspect of mucosal response is considered time-independent.

#### 3.1.1. Linear elastic

Linear elasticity is a simplified version of a more generalized nonlinear elasticity which has formed a branch in continuum mechanics. This constitutive model governs reversible behaviour of a material which is indicated by a straight stress–strain response curve with a constant elastic modulus. When subjected to sufficiently small stresses, nearly all solid materials can be represented by linear elastic constitutive equations (equation (3.1) for an isotropic case), which are relatively easy to solve. The linear elasticity model is thus the best known and most widely used theory in biomechanics.3.1
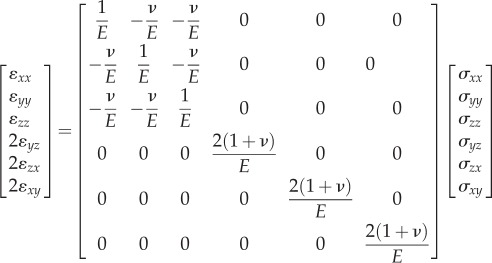


At the early stage of exploring the stress–strain relationship of the mucosa, the experimental reports showed a wide range of possible compressive elastic moduli from 0.06 to 8.89 MPa when using a ‘dead’ weight or an instant load [29,35,41,63,64]. Meanwhile, there were several other relevant findings. Firstly, the mucosa is generally stiffer under tension than compression, showing elastic moduli ranging from 0.91 to 11.12 MPa [63]. Secondly, it has anisotropic responses under both tension and compression [63]. Lastly, both mucosa thickness and elastic moduli can vary considerably in the same subject [35] and between individuals [64]. Compared with other oral soft tissues, such as the periodontal ligament (PDL), the oral mucosa exhibits lower stiffness [65] and the tendency to deform more easily, with a difference of more than three times in the tissue displaceability relative to the PDL [66].

During modelling of linear mucosal elasticity and the associated responses with dental prostheses (e.g. complete and partial dentures, dental posts, bridges and implants), a broad range of elastic modulus values have been adopted in research, often by assumption. Initially, owing to lack of sufficient experiment data, the skin properties (19.6 MPa) for being another typical soft tissue were adopted [67], and this assumption was accepted in two other studies [68,69]. Another two elastic modulus values (10 MPa [70] and 5 MPa [71]) were first reported in non-English journals. Note that both such assumptions gained considerable acceptance, such as [72–76] for the former and [77–79] for the latter. To simulate the effects of different mucosa resiliency to compression, elastic moduli of 340 MPa and 680 MPa were assumed for the hard and medium mucosa, respectively, compared to the soft mucosa (1 MPa) [80–83]. At the other extreme, a very low elastic modulus of 0.1 MPa was also assumed [84,85], and so was 0.68 MPa [86,87] in literature.

There were also elastic moduli derived from experimental observations. A typical value of 1 MPa was derived from the experiment by Picton [66], and adopted in several finite-element analysis (FEA) studies [88–97]. Similarly, other values between 1 and 5 MPa were reported experimentally [63,64] and were adopted for simulations [98–106].

**Figure 2.**
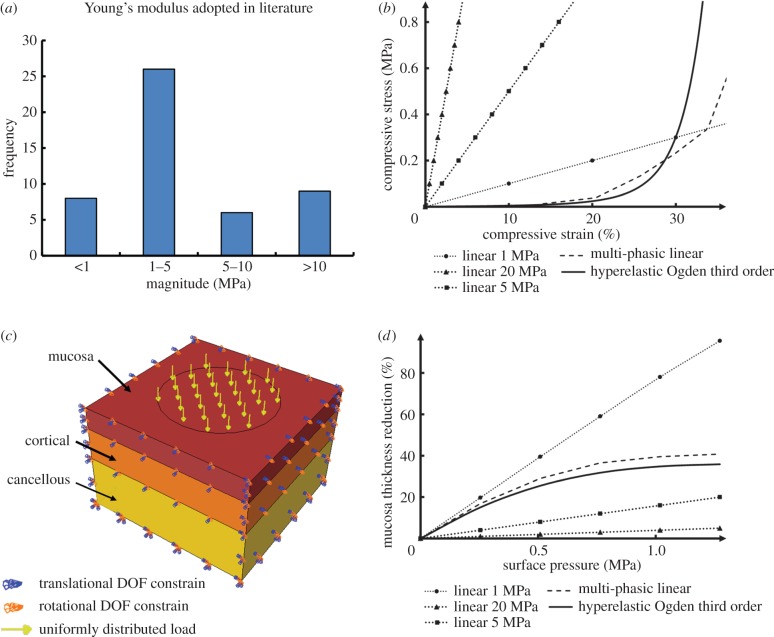
(*a*) The frequencies of different linear elastic moduli adopted in existing FE studies; (*b*) a simplified model to present a unit of mucosa–bone structure; (*c*) the compressive stress–strain relationships between different material models (linear elastic, multi-phasic elastic and hyperelastic); and (*d*) the maximum mucosa thickness changes in the different material models of mucosa under increasing loads up to 100 N in the test model.

All these linear elastic models from the literature assumed linearity with homogeneity and isotropy of the mucosa, although it has been anatomically demonstrated as a heterogeneous and anisotropic composite material [63], responding to mechanical loading in a complex nonlinear manner [107]. Despite the over-simplified mechanics and limited supporting biological evidence, linear elasticity has its advantages in providing a simple and direct prediction of the mucosa's instant responses. A simplified elastic model is also preferred for the sake of the computational efficiency [108]. Therefore, the linear elastic material model has been extensively adopted in a range of studies and has achieved wide acceptance, especially in the clinical field. Nevertheless, in such a simplified material model the elastic modulus varies over an enormous range from 0.1 to 680 MPa, which consequently alters the soft-tissue behaviour dramatically. Figure 2*a* summarizes the frequencies of different linear elastic modulus values appearing in previous studies, and figure 2*b* shows some examples of linear elastic models with the moduli of 1, 5 and 20 MPa.

#### 3.1.2. Biphasic and multi-phasic linear elasticity

Previous studies have shown that the reduction of mucosa thickness was not proportional to the increase in loading [109]. With further increased compressive loads, the mucosa becomes more resilient to deformation, suggesting an increasing elastic modulus with higher pressures [107]. The histological analysis indicated that the nonlinearity may have resulted from microstructural deformations, such as buckling and loss of space in the fibrous network and epithelium [32], leading to different mechanical behaviours at different levels of strain. Consequently, the simplest linear elastic model could not address the nonlinearity of the mucosal response properly [62,110].

A biphasic linear elastic model was developed by using two moduli for approximating a nonlinear stress–strain curve, thereby addressing the change in the initial and subsequent moduli in a path-dependent manner. The switching between these two moduli is determined by mechanical stress (equation (3.2), where *n* is the number of phases, *n* = 2 for such a biphasic model), strain, or strain energy at a typical conversion point. The approach captures more features of the tissue responses, without substantially increasing computational cost. The effectiveness of such a bilinear material was verified using animal studies along with the other oral soft tissue, such as PDLs [111], and it was applied in the associated FEA [112]3.2
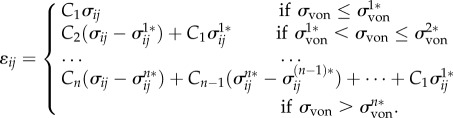


While considering the modulus rise with deformation strain, the biphasic linear elastic model still remains relatively simple and primitive; and few studies of relevance to mucosal responses have adopted this material model. Instead, a subsequent multi-phasic linear elastic material model (as the generalized form in equation (3.2)) was developed, which was capable of capturing a more precise loading path for the mucosal deformation [113] (e.g. the dash line in figure 2*b*). The multi-phasic linear elastic material model has a series of path-dependent elastic moduli and corresponding conversion points at different loading extensions, to better imitate the nonlinear behaviour. This material model was derived based on the *in vivo* results of mucosal responses in the literature [107], by using six von Mises (VM) stress values as determinants of the conversion path, and the compressive response matches reasonably well with the *in vivo* measurements. This model enables a balance to be made between accuracy and computational efficiency, as the true nonlinear analysis requires a much larger number of loading steps with a substantial time penalty. With the increasing number of elastic phases, the stress–strain curve approaches the real nonlinear more closely, and the computational time rises in turn with more iterations.

#### 3.1.3. Hyperelasticity

Even with a multi-phasic linear elastic material model, the exact nonlinear elasticity cannot be entirely reproduced, as segmented straight lines do not represent the true equilibrium path. A hyperelastic material (also so-called ‘Green’ elastic material) requires a constitutive model that derives the elastic response from a strain energy density function, providing continuous stress–strain interpretation to modelling of material nonlinearity. It has been commonly applied in the mechanics of rubber-like materials, and the similarity to biological soft tissues has recently attracted notable attention [114]. These types of material models respond elastically (reversibly) under very large strains, which is exactly what a biological soft tissue does under both normal and pathological conditions [115].

Hyperelastic material modelling starts with the formulation of a potential energy function based upon scalar strain. The strain energy potential defines the strain energy stored in the material per unit of reference volume (volume as in the initial configuration) as a function of the strain at a typical point in the material. Such functions can be dependent either on strain tensors of a nonlinear deformation field, on the invariants of these strain tensors, or even directly on the principal stretches. Simply speaking, the hyperelastic material describes the stress–strain relationship using a continuous function rather than one or a series of elastic constants, generating a true nonlinear map of behaviour.

Hyperelastic material models can be generally classified into two categories, mechanistic (micro-mechanical) and phenomenological (macro-mechanical) [116]. The former is directly derived from statistical mechanical arguments of the underlying material structures or idealized network, such as cross-linked polymers. Arruda–Boyce and neo-Hookean are the two such models in this category [116]. The mechanistic category is intrinsically tied to higher computational costs for its homogenization procedures, where the micro-mechanical details are associated with the macroscopic mechanical behaviour by using the governing parameters. Despite this profound basis, the requirements for understanding the structural composition and associated behaviours are extremely difficult in such mechanistic models, and often remain unclear or understudied for most biological tissues.

The phenomenological category, on the other hand, aims to link the functions to the direct empirical observations of phenomena, thereby matching with the fundamental theories. The functions in this category include Fung, Mooney–Rivlin, Ogden, polynomial, Saint Venant–Kirchhoff, Yeoh and Marlow [116]. Ogden, being a popular type, can be expressed as in equation (3.3), in which 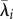 are the deviatoric principal stretches obtained from the principal stretches, *N* is the order of the fitting equation, *J*^*ɛl*^ is the elastic volume strain, and *μ_i_*, *α_i_* and *D_i_* are the parameters for such a hyperelastic model.3.3
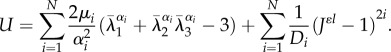
Compared to the stringent conditions required for the mechanistic category, the phenomenological models present distinctive advantages. The approach of fitting hyperelastic models to experimental data has been addressed in a number of textbooks [117,118] and mechanics studies [114,119–121], which has been adopted for modelling several different types of soft tissues in the human body, such as ligaments [122,123], meniscus [124], skin [125], oesophagus [126] and the oral PDL [127,128]. Recently, Winterroth *et al.* [129] characterized the nonlinear elastic property of engineered oral mucosal tissues by using scanning acoustic microscopy and fitting data to the first-order Ogden strain energy potential function (equation (3.3), where *n* = 1). Recent developments in computational power and numerical techniques have enabled more realistic models of tissue behaviours [97,113,127,130,131]. Nevertheless, using the hyperelastic material model to simulate the native oral mucosa response remains preliminary, which may be due to the requirements of incorporating its high nonlinearity and anisotropy [132,133]. Recent progress on modelling of anisotropic hyperelasticity in other soft tissues has been documented in several studies [134–136], but limited information, in either clinical data or experimental measurements, is available for the oral mucosa with highly integrated heterogeneous anatomical microstructures and complex physiological responses. Only few recent studies [137–140] developed the hyperelastic model based on *in vivo* measurements. Figure 2*b* includes an example of the hyperelastic material model (Ogden third order) derived from the clinical data reported by Kishi [107].

#### 3.1.4. Comparison

To illustrate the differences between the above-mentioned elasticity models, a simple three-layer block (representing mucosa, cortical and cancellous bones) is adopted herein to simulate the local mucosal responses under uniformly distributed compression over an area of 10 mm in diameter (figure 2*c*). A mucosal thickness of 2 mm is assumed here based on average clinical measurements [8]. Periodic boundaries are prescribed to the surrounding sectional planes to simulate the tissue continuity with the neighbours, and a full constraint was assigned to the bottom of the block. The load on the top surface was ramped from 0 to 100 N in this model.

The material properties for the bony structures are considered isotropic and homogeneous, following previous studies in the literature [127] in order to set a baseline. All three static elastic material models (linear, multi-phasic and hyperelastic) were considered for the mucosa. These three linear elastic moduli are adopted at 1 MPa, 5 MPa and 20 MPa, respectively, to simulate low, medium and high stiffness in the most accepted range of literature values. The multi-phasic model was adopted as developed by Kanbara *et al.* [113]. The hyperelastic material model (Ogden third order) is derived from the empirical data by Kishi [107]. Poisson's ratio is set to be a constant of 0.3 for all material models so as to focus the differences entirely on elasticity values and material constitutive models. Figure 2*d* plots the percentage change of the maximum mucosa thickness against the increasing loads under different material models.

### 3.2. Viscosity and permeability

Accompanying the instant elastic responses, the oral mucosa also exhibits a dynamic response over the time under loading and upon unloading, interpreting as creep and delayed recovery [21,65,141]. It is believed that, not only the interstitial fluid and blood, but also the fluidic components within the mucosa matrix considerably contribute to this time-dependent behaviour [142]. Both the fluidic viscosity and permeability influence the dynamic response, but the former has been better studied than the latter based on the number of publications available. Being a complex composite material, neither the viscosity nor the permeability alone represents the mucosal characteristics; they are concurrent with the elasticity, either linear or nonlinear. This section will focus on two material models, viscoelastic and porous elastic (poro-elastic).

#### 3.2.1. Viscoelasticity

The time-dependent response was firstly quantitatively illustrated as the viscoelastic property by a histometric analysis conducted on dogs in the time domain [141], which suggested that, apart from the elastic response, there was a viscous component in this fluid-rich material. The viscoelasticity manifested four stages of behaviour under loading and unloading, namely instant deformation, creep, instant recovery and delayed recovery.

Upon immediate loading, the instant elastic deformation (first stage) takes place as elucidated by its elasticity, with a relatively less notable viscous response in such a short time. The following creep at constant load (second stage) can last for more than 6 h with the trend continuing [141], sometimes for days. The extent of the creep can vary from 4 to 30% of the total mucosa thickness [40,107,109], and gradually slows down after 1 min [107]. The ‘elastic’ modulus after the creep stage settles usually after 1 h, which is called the ‘steady’ modulus, and it can however vary from 0.04 to 2.35 MPa [35,41,107,109,143]. Upon unloading, some proportion of the elastic deformation recovers (third stage), typically from 46 to 91% of the total mucosa thickness, which is also dependent on the loading history, including magnitude and duration, in a nonlinear manner [6,66,109]. Similar to creep, the delayed viscous recovery (fourth stage) continues for much longer than the instant recovery, and may reach 70–90% of the initial thickness [41,109]. Compared with the PDL [65,66], the protracted recovery that was observed in the mucosa, which could take more than 1 h to complete, while it was only 1–2 min for the PDL. With increasing loads, these differences became significant, suggesting a more considerably viscous behaviour in the mucosa.

Several factors can affect the viscoelastic response and are attributed to the physiology of incorporated biofluid. The mucosa in the elderly population often has more significant viscous behaviour, especially the prolonged time and reduced rebound with delayed recovery [6]. It was suggested this arose because of the reduced amount of elastin and the greater capability of maintaining fluid in the mucosa with patient age [21]. Increasing contact areas generally leads to stiffer mucosal responses [6,107,109], and higher loading rates also have similar effects [35,66,109]. Male subjects were found to exhibit a stiffer mucosa response with slower recovery than female subjects [29], and it was suggested that female subjects usually have a thicker mucosa than male subjects [6].

**Figure 3.**
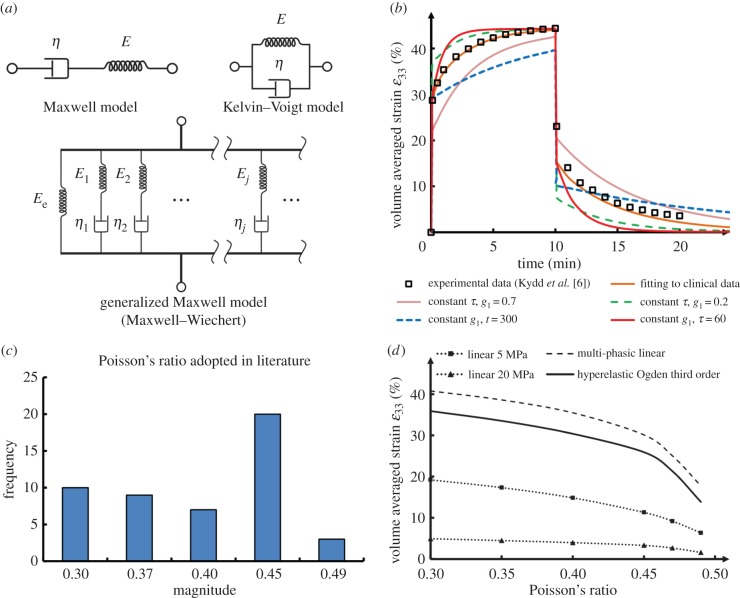
(*a*) The schematic diagrams of common viscoelastic material models; (*b*) the viscoelastic responses of different parameters in the test model, compared to the clinical data; (*c*) the frequencies of different Poisson's ratios adopted in existing FE studies; (*d*) the volume-averaged strain responses by the different Poisson's ratios of mucosa under 100 N in the test model.

The most fundamental material model for viscoelasticity has two components as observed in experimental studies, elasticity and viscosity [144], which can be modelled in series (known as the Maxwell model, figure 3*a* upper left) or in parallel (known as the Kelvin–Voigt model, figure 3*a* upper right). A materials' elasticity can be a path-dependent factor following Hooke's Law just like a spring, and the viscosity exhibits the time-dependent effect like a dashpot.

In the literature, there are only few reports on the usage of viscoelastic models for mucosa. Two of the early studies [145,146] assumed the orthotropic mucosa properties in a simplified two-dimensional finite-element model by taking a standard linear solid of a Kelvin–Voigt and Maxwell model in series, with the elasticity of *E*_1_ = 1.1 MPa, *E*_2_ = 1.2 MPa and the viscosity *η*_1_ = 18 MPa s, *η*_2_ = 250 MPa s. Other researchers [7,147] assumed an isotropic, homogeneous and linearly elastic body under isothermal conditions, and attempted to use an exponential function (equation (3.4)) [148]. In this equation, the modulus is dependent upon time (*t*) and determined by two parameters, the initial modulus (*E*_0_) and the relaxation time (*τ*). By matching the numerical model with the clinical data, the initial modulus was determined through a reverse-engineering approach at 0.083 ± 0.020 MPa, and the relaxation time is 503 ± 46 s [7,147].3.4
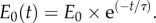


These two-component systems are the simplified version of the generalized Maxwell model (or Maxwell–Wiechert model, figure 3*a* lower), in which several Maxwell elements (spring plus dashpot) are assembled in parallel to accommodate more complex relaxation and creep behaviours.

Prony's method is often used in the interpretation of the experimental data, to derive the coefficients for the Prony's expansion of multiple exponential terms (equation (3.5), for relaxation modulus) [149,150]. It should be noted that *G* in this equation represents the shear modulus, but it can also be tensile–compressive modulus *E*, or bulk modulus *K* when needed, *τ* is the relaxation time. Often, the relaxation coefficient (*g_i_*) is normalized against the modulus as in equation (3.6).3.5
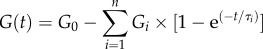
and3.6
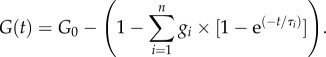


Besides the time domain, another approach is to study and model the viscoelasticity in the frequency domain, by using dynamical mechanical (DM) testing and magnetic resonance elastography (MRE) [151–153], where a small oscillatory stress is applied and the resulting strain is then measured. This approach expresses the viscoelastic properties by using the complex modulus (equation (3.7)).3.7
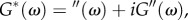
where ‘i’ is the imaginary unit, *G*′ and *G*″ are the storage modulus (elastic) and the loss modulus (viscous), respectively. The stress and strain are in phase for the purely elastic materials, generating the immediate response of one caused by the other, as indicated by 0 in the second term. By contrast, the purely viscous material has a 90° phase lag in strain response. Viscoelastic materials behave somewhere between these two extreme types of materials. The two complex modulus parameters were determined as 2.53 ± 0.31 MPa (*G*′) and 0.90 ± 0.22 MPa (*G*″) by *in vivo* MRE [154]. The impact of fluid amount in the mucosa was also verified under DM [143]. While this approach has been applied to numerical modelling of the PDL [155], there has not been any report on the mucosa to date.

#### 3.2.2. Porous elasticity (poro-elasticity)

In contrast to the viscoelastic material model that assumes a homogeneous material, the porous elastic model considers the mucosa as a two-phase material, consisting of the solid porous matrix (e.g. collagen) and the ground (fluidic) substance (e.g. watery solutes) [52]. The interstitial fluid for the mucosa is allowed to flow from a stressed region to the unloaded neighbour regions, and the permeability of the structure changes under different mechanical conditions, decreasing or increasing the flow. This fluidic behaviour is described by Darcy's Law (equation (3.8)), in which *Q* is the total discharge rate (usually in mm^3^ min^−1^), *A* is the active area, *h* is the specimen thickness and Δ*P* is the pressure difference to drive the flow. The permeability *k* in equation (3.8) is porosity-dependent (equation (3.9)) and is affected by the void ratio *e* at a certain time instant [156]. At zero strain, *k*_0_ is the virgin permeability at the initial void ratio *e*_0_. *M* is a dimensionless constant.3.8
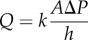
and3.9
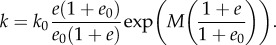


Current research interest regarding mucosa permeability lies in drug delivery through oral tissues [157,158], and the permeability examined in the literature was mostly for the absorption from the external space through the mucosa (perpendicular to the mucosa). Owing to the structural complexity and the difficulty in preserving mucosa integrity, the permeability (parallel to the mucosa) that defines internal fluid flow has not been well studied. For other oral soft tissues, e.g. the PDL, *in vivo* tests [159] have been performed to investigate the role of interstitial fluid on its mechanical response, and a computational model of porous hyperelasticity (nonlinear elasticity) has been developed to match with the experimental results [160]. The initial permeability *k*_0_ and the dimensionless constant *M* were found to be 8.81 × 10^−9^ mm^2^ and 14.2, respectively, which provide some insight for further studies on the mucosa.

#### 3.2.3. Comparison

As some fundamental data for the mucosa are not yet available in literature for incorporating into a porous elastic material model, this review focuses on the dynamic differences in the viscoelastic model, by varying the viscous terms. A Prony series is adopted as a general approach to deriving the viscous response of soft tissue from clinical data by the least-square method [149]. Based on the creep data reported by Kydd *et al.* [6], a first-order Prony series (one exponential term, equation (3.10), instant elastic modulus *E*(*t*)) provides sufficient fit (strain error < 1%). The linear elastic constant, *E*_e_, is inversely determined at 0.083 MPa assuming Poisson's ratio at 0.3, similar to some early reports [7,147]. The normalized relaxation coefficient, *g*_1_, is found at 0.527 (or 0.044 MPa for the absolute value with the determined elastic modulus), and the time constant, *τ*_1_, is 90.6 s.3.10
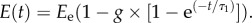


We adopted these inversely determined parameters for the same model used in §3.1, and tested this material model under a constant loading of 50 kPa (equivalent to the average contact pressure under a common denture base with an occlusal load of 150 N) [7]. The volume-averaged strain under the loading area along the loading direction (*ɛ*_33_) is plotted against time (brown solid line, figure 3*b*), showing 10 min of creep and 10 min of recovery. The clinical data [6] are also included as shown by black rectangles for comparison.

Upon varying one of the two parameters, we can compare the variation in mucosal responses. With constant *τ*_1_ (90.6 s), the higher normalized relaxation coefficient *g*_1_ at 0.7 (pink solid line) implies an increased viscous response than the elastic component, whereas the lower *g*_1_ at 0.3 (green dash line) is opposite. At constant *g*_1_ (0.527), the time constant *τ*_1_ at 60 and 300 s indicates faster creep (blue dash line) and slower creep (red solid line), respectively.

### 3.3. Poisson's ratio

Poisson's ratio is the other fundamental mechanical property similar to the elastic modulus, which defines the volumetric response of the mucosa to mechanical loading. It is the tendency to resist a volumetric change when the material is deformed; and it is often defined by the negative ratio of the transverse strain to the longitudinal strain. Under compression, material tends to expand sidewise along the perpendicular directions to the loading direction; while under tension, it then tends to shrink sidewise. As another mechanical property, Poisson's ratio indicates the compressibility of material, and the value of 0.5 indicates a perfectly incompressible material. Thus, the volumetric behaviour of the oral mucosa can be determined by its Poisson's ratio.

As the oral mucosa is a nonlinear and heterogeneous composite material, this volumetric response is more appropriately considered as ‘the apparent Poisson's ratio’ or ‘Poisson's effect’, to reflect the homogenized behaviour generated by all the individual components involved. Thus, the term ‘Poisson's ratio’ used in this review is for brevity and common acceptance in elastic materials.

Compared to the exhaustive investigation conducted on the mucosa stress–strain relationship (elasticity), few reports are available regarding its lateral responses, or its compressibility, with surrounding neighbour tissues involved. One of the primary reasons is the difficulty in measuring the lateral response. The highly complex and continuous anatomic morphology makes direct measurement *in vivo* difficult (if not impossible), and the mucosa acts as a unit from the surface epithelium to the sub-surface periosteum bonded to the bone, which prevents *ex vivo* loading to break its integrity. There are some non-invasive *in vivo* techniques to measure the displacement/strain responses in soft tissues but these are somewhat limited; they are termed elastography (and include ultrasound elasticity imaging, magnetic resonance elasticity imaging and tactile imaging) [161–164]. These image-based techniques can monitor the lateral motion under constant compression or dynamic vibration along the axial motion. In addition to the benefits of being non-invasive, the accuracy significantly relies on the image resolution and noise deduction procedures. So far, the only application of elastography to the oral mucosa was documented by Cheng *et al.* [154] on its elastic modulus, but no information was reported on Poisson's ratio or lateral response. Apart from the primary technical issues, the other reason is perhaps the insufficient awareness of the importance of Poisson's ratio. In fact, the discrepancy of different Poisson's ratios was claimed as a non-critical factor for its response in the literature [29].

Without sufficient experimental data, most finite-element studies have made assumptions of Poisson's ratios based upon the knowledge gained from other soft tissues. One typical value of 0.3, adopted from skin [67], has been widely accepted for static linear elastic studies [68,69,84,85,92–95] and dynamic viscoelastic analysis [7,147]. Another two values often appearing in literature are 0.37 [108] and 0.4 [72], derived from earlier experimental studies [66,70], and have gained wide acceptance [74,76,80–83,88–90,96–99].

Biological soft tissues are often considered as ‘incompressible’, and being one of them, the mucosa was also assumed to have higher Poisson's ratios to simulate the low compressibility or non-compressibility (perfectly incompressible). The values of 0.45 [75,80–83,86,87,91,101–104,106,165] and above [77,78,79], or even 0.5 [29] have been suggested for finite-element study purposes. Apart from the constant Poisson ratio, a series of multi-phasic Poisson's ratios have been adopted by Kanbara *et al.* [113], in which Poisson's ratio increases with VM stresses at the conversion points from 0.3 to 0.49. In conclusion, a range of Poisson's ratios from 0.3 to 0.5 have been adopted in previous studies, and the frequency in the literature is summarized in figure 3*c*. A very recent study adopted an inverse method of determining the apparent Poisson ratio in the oral mucosa from *in vivo* contact pressure measurements, and based on this patient-specific case, the values were found to be 0.402 [139].

Some soft tissues, such as the oesophagus [166], pulmonary airways [167], blood vessels [168] and even tumours [169], demonstrate their abilities of buckling and forming surface wrinkles under compression, contributing to both physiological and pathological developments. Such behaviour is induced not just by the low compressibility or incompressibility, but also by the combining effects of their geometrical features (tubular shape) and low elasticities [170–172]. The anatomical structure of the oral mucosa is different to these types of soft tissues. As illustrated in §2, the mucosa is bonded to the bone beneath via a mucoperiosteal layer, rather than the mucosal–submucosal–muscular structures in the tubular soft tissues. Therefore, the morphological instability of the oral mucosa is not so obvious, and yet there are no studies investigating its surface wrinkle formation, leaving the potential to explore the physiological meaning of this for future studies.

Nevertheless, to illustrate the effects of Poisson's ratio on mucosal responses, the same model used in §3.1 is tested with Poisson's ratios ranging from 0.3 to 0.49, with linear elastic (*E* = 5 and 20 MPa) and hyperelastic (Ogden third order) material models, under a constant load of 100 N. The volume-averaged strain is plotted in figure 3*d* against the increased Poisson's ratio values. Clearly, Poisson's ratios affect the mucosal response in a nonlinear manner, where the higher the Poisson ratio, the less deformable the tissue.

### 3.4. Friction coefficient

The oral mucosa, being a protective layer over the residual ridge, does not only sustain compressive loading, but also the surface shear induced by the friction beneath the dentures. The prevalence of mucosal lesions associated with denture wearing is well known. Acute or chronic reactions to the mechanical injury can be caused by both microbial denture plaque and constituents of denture materials [173]. Most of these denture-induced symptoms, such as traumatic ulcers, angular cheilitis, irritation hyperplasia and keratosis, are related to the frictional loading on the mucosa and are hard to cure [174–176].

Understanding the interactive response between the denture and the supporting mucosa is critical to prevent soft-tissue injuries, and the associated occlusal load transmission requires correct determination of nonlinear elastic contact. This interactive response can be related to the friction coefficient, which differs significantly among subjects, depending on their oral physiological conditions and denture materials used [177].

The variability of saliva generation alters the friction coefficient, thereby affecting the contact conditions [178]. Xerostomia (known as dry mouth) is one of the most common problems in the elderly edentulous population, associated with reduction of saliva production, which has been shown to have a severe impact on denture usage, leading to membrane stomatitis [77,177,179,180]. In experimental studies, high friction coefficients between 0.3 and 0.4 were reported with ‘dried’ surfaces (hydration index closes to 0, to simulate xerostomia) [178,181], whereas a low value around 0.02 was reported for well-lubricated conditions [181].

With the same oral condition, the friction coefficient can also change between different denture materials. A material with higher wettability will be more likely to form a superior lubricating layer between the supporting mucosa surface and the denture base, thus protecting the surface tissue by reduced friction. Seven types of common denture liner materials were tested *in silico* in literature [182]. Under dry conditions, the friction coefficient was between 0.35 and 0.97; after being wetted in a warm water bath, the friction coefficient dropped to between 0.24 and 0.90. Acrylic resin material was found to have significantly better wettability than silicones [183], and the friction coefficient decreased drastically when wet [182].

Clinically, no effective *in vivo* approach has been reported for measuring the friction coefficient of individual patients in literature, and the only friction coefficient inversely determined from *in vivo* contact measurement was 0.213 most recently [139]. Meanwhile, owing to the complexity of the nonlinear contact simulation, the results of such finite-element studies are somewhat diverse. By comparing the linear and nonlinear contacts under the denture base, a finite-element study found that while the difference was less than 20% in terms of the magnitude of the VM stress in the mucosa and claimed to be insignificant [184]. Other studies have adopted either fully bonded, fully tied or other linear contact mechanism between the denture and the mucosa, to simulate a linear transmission of occlusal forces [78,83,86,87,92,99,100,102,106]. On the other hand, while incorporating this nonlinear mechanism, most studies adopted different frictional coefficients ranging from 0 (frictionless) to 0.75 (penalty contact) [76,77,84,85,88,90,91,93,97,101,147,178,180,185].

**Figure 4.**
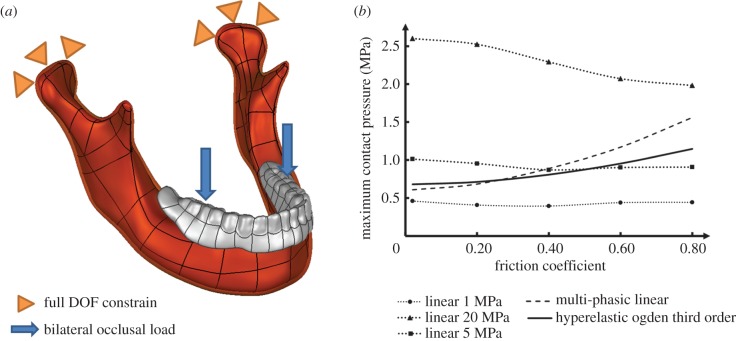
(*a*) The schematic diagram of the finite-element model in the friction coefficient test; (*b*) the maximum contact pressure against increasing friction coefficients in different material models.

Nevertheless, there has been no systematic study on the effects induced by different friction coefficients, and this review will test the common range reported in literature, from 0.02 to 0.8, for both linear elastic (elastic modulus at 5 and 20 MPa) and hyperelastic (Ogden third order) material models as used in §3.1.3. The interactive reaction is highly dependent on the surface morphology of the interface; therefore, a simple three-dimensional jaw model is constructed from the CT images. The complete denture is made of acrylic containing BaSO_4_, to impart radio-opacity, with an elastic modulus of 2.67 GPa and a Poisson's ratio of 0.167 [137,186]. A pair of bilateral occlusal loads equivalent to 60 N is assigned to the vicinity of the first molar, along the tooth root direction (figure 4*a*) [137]. As the primary indication to the pathological consequences, the maximum contact pressure of the mucosa surface is plotted in figure 4*b* against the frictional coefficient. The linearly elastic material models show either marginal differences or a decrease in the maximum contact pressures, with increasing friction coefficients, which obviously do not match the clinical observations [178,181]. In this figure, the path-dependent material models, multi-phasic elastic and hyperelastic, show gradually increasing maximum contact pressures with increasing friction coefficients, which appears to be more realistic to clinical measurements [181].

## 4. Clinical implications

All biomechanical models of the mucosa serve the purpose to interpret, analyse and predict the various biomechanical aspects of the mucosal responses to dental prostheses, to optimize treatment outcomes with minimum side effects to patients. This section illustrates some common clinical concerns and links them to the biomechanics for identifying specific insights relevant to dental prosthetic design and treatment planning.

### 4.1. Tissue stimulus

Often mechanical bodies experience more than one type of mechanical stresses (e.g. normal and shear) along different directions, and a general expression of these stresses can be defined by the Cauchy stress tensors (equation (4.1)). To assess the collective effect of these individual components on biological variations, several scalar forms can be computed from the Cauchy stress tensor, such as the VM, Tresca and maximum principal stresses4.1
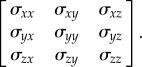


Of these scalar forms, the VM stress (equation (4.2)) has been most widely applied in the FEA for engineering problems, suggesting that the yielding of material occurs once the second deviatoric stress invariant reaches a critical value. In biomechanics, it is often known as the equivalent stress, its applications to dental implants and other metallic prostheses (such as some parts of the partial removable denture, the metallic sleeve/bar within overdentures) has been well recognized [82,86,87,130,187]. With assumptions regarding homogeneity and isotropy, the application of such an equivalent stress has been extended from metallic materials to both cortical and cancellous bones for its strain energy relevance [188–191].4.2
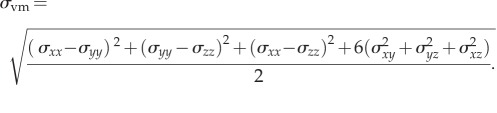


A histological study on the anatomy of the mucosa has revealed it as a complex structure with a large number of channels and vessels [46]. The interstitial fluid filling this porous structure can flow to the neighbouring mucosa under compression and transmit loads through a highly vascularized network embedded inside the mucosa [21,32]. This fluid-induced stress over any nominal internal plane is equal in magnitude and always directed perpendicular to this plane, regardless of its orientation. This isotropic stress status is known as the IFP or mechanically hydrostatic pressure (equation (4.3)) within the mucosa, which indicates the functional pressure inside the tissue. Different to the VM stress, the hydrostatic pressure is related to the first stress invariant as follows:4.3
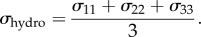


The hydrostatic pressure from animal studies varies across different locations in the oral mucosa [192–194]. In rats, the highest IFP of 1.97 kPa was found at the hard palate, and the lowest ones were found at the alveolar mucosa and the free gingiva at 0.48 and 0.31 kPa, respectively. Around the attached gingiva, the pressure can vary from 1.14 to 1.23 kPa. The hydrostatic pressure can increase if there is an inflammatory response [195], which may occur after denture insertion [21,22] and consequently compromise mucosa permeability [158,196]. Being one of the most important aetiological factors to denture-induced symptoms [9–14], excessive IFP (or hydrostatic pressure) can reduce blood circulation and even temporarily cause localized ischaemia [26,47–49], accompanied by pain and discomfort [65]. Such prolonged excessive pressure may lead to the destruction of the supporting bony tissues, known as residual ridge resorption [8–10].

**Figure 5.**
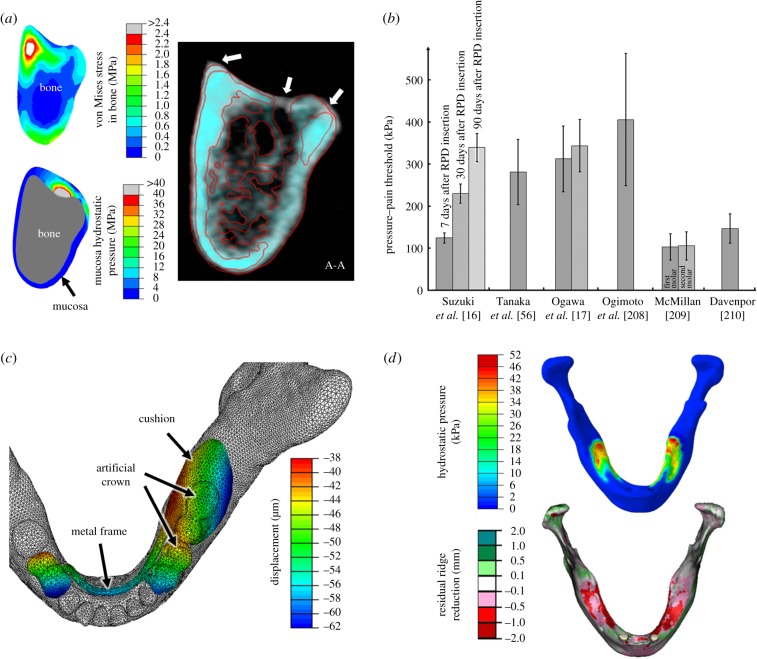
(*a*) The distribution patterns of the VM stress in bone and the hydrostatic pressure in mucosa compared to residual ridge resorption under CT (white: before denture insertion; cyan: 1 year after denture insertion); (*b*) the PPT thresholds determined using the clinical data from the literature; (*c*) the vertical displacement of a removable partial denture under an occlusal load of 60 N on the first molar and (*d*) the mucosa hydrostatic pressure pattern versus the residual ridge height reduction.

To investigate mucosal responses to external loading, such as denture insertion, the hydrostatic pressure determined from FEA provides a meaningful indication of possible internal biomechanical changes [197–200]. Figure 5*a* compares the distributions of the VM stress in the bone and the hydrostatic pressure in the mucosa to examine their relevance to residual ridge resorption measured from two sets of CT images over 1 year duration. The white mask in the CT image is the pre-insertion status of the patient, and the cyan mask is 1 year post-insertion. The white triangles indicate the most severe locations of bone resorption, which is obviously better correlated to hydrostatic pressure distribution.

While fundamental knowledge concerning hydrostatic pressure has been well studied in fluid statics problems, its application to biological tissues is gradually increasing and being recognized over a wide range of anatomical components, such as stomach, heart, liver, lung, ligament and cartilage [200–204]. For the oral mucosa, it has been used to evaluate the possibility of tissue remodelling driven by the occlusal loads during tooth eruption under the combined stimuli of intermittent tongue, lip and cheek actions [205]. In the other oral tissue, the PDL, the hydrostatic pressure has also been shown as a key mechanical stimulus for remodelling in the surrounding bony structure during orthodontic treatment [127], as well as the accompanying root resorption [197,206]. If the hydrostatic pressure in the PDL exceeds the capillary blood pressure, partial or complete collapse of the capillaries may occur just like in the mucosa. The distributions of hydrostatic pressure matched well with the clinical observations of residual ridge reduction [137,138,140,207].

Hydrostatic pressure also plays a role in predicting the outcome of removable denture treatments, which is closely associated with both mechanical and physiological functions of the soft tissues beneath denture bases. Mechanically, the mucosa acts as a buffer or cushion to distribute the mastication loading from the denture to the supporting bone. Physiologically, the blood vessels provide nourishment to the supporting bone of the denture foundation. A denture that mechanically abuses the subjacent soft tissues hinders the physiological functions of these tissues. On the other hand, any systemic condition that unfavourably affects the physiological function also influences the mechanical capabilities of the tissues, thereby jeopardizing the outcome of such denture treatments [62].

### 4.2. Pressure–pain threshold

The sensation of pain is the most direct indication of a maladaptive denture to the supporting mucosa, and it is the most common and critical issue affecting denture function [17]. While the biochemical pathway of triggering the pain is not yet fully understood [208], previous research has revealed that high contact pressure can cause pain in the mucosa [209–211]. To clarify this statement, the contact pressure here refers to the load borne perpendicularly on the mucosal surface, rather than the internal hydrostatic pressure. A PPT has been proposed as a measure of the lowest pressure that causes pain, which links the objective stimulus (pressure) to the subjective response (pain) in a quantitative fashion. A pressure algometer is a common technique to measure the *in vivo* PPTs, and its validity and reliability have been verified in the literature, showing positive and consistent associations in clinics [208,212].

Several studies have been carried out to investigate the PPT (figure 5*b*), and it was found to vary from 102 to 405 kPa [17,56,208–210,213]. There are several factors affecting the PPT, including mucosa thickness, morphology, location, age, loading rate and loading history. Patients with a thin mucosa covering sharp bony ridges are more likely to have a lower PPT than those with a thick mucosa over a flat bone surface under a denture base [14,109]. The loading locations, such as the palatal, lingual and buccal mucosa, have their different morphologies, thickness and anatomical features, leading to the various PPTs observed in clinics [56,208,209,213]. The viscous responses associated with interstitial fluid are reflected in both the loading rate and loading history as discussed in §3.2. Slower loading rates generally result in lower thresholds, as the fluid has more time to flow into unstressed neighbouring areas before building up substantial resistance to internal deformation [208,209,212]. By contrast, a faster impact stiffens the tissue and develops a higher local pressure [7,21,147]. The pain tolerance can ramp up with increasing loading duration, and the extent of the recovery of the mucosa affects the subsequent PPT [16,17].

All these factors above are reflected in the biomechanical responses of the mucosa [14]. Simplified mucosa material models (e.g. linear elastic) often find that the denture-induced pressures [92,214] are below the measured pain thresholds, which is contrary to the clinical observations [215]. Correctly established finite-element models can provide objective diagnostic criteria of the surface contact pressure for predicting the discomforts induced by denture treatment. Furthermore, the internal hydrostatic pressure can be used from the transmission of contact pressure through the mucosa, which allows further insights to be gained regarding biomechanics triggering the pain sensation.

### 4.3. Tissue displaceability

Some dentures are not fully supported by a single type of tissue, and they more often distribute occlusal loads unevenly to multiple supporting tissues, such as the teeth (including the PDL), mucosa and bone around an implant [130]. The tissues have quite distinct material behaviours, which alter denture deformation in a complex manner. Removable partial dentures and implant-retained overdentures are some typical examples, which are not entirely tooth/implant supported but also supported by mucosa and bone. These differences of displaceability lead to varying denture/tissue deformation in both directions, along and across the residual ridge. As an example, figure 5*c* shows the displacement of a removable partial denture under occlusal loading (60 N on the first molar of the denture).

Compared with complete dentures, the teeth-supported partial denture and the implant-retained overdenture have a much stiffer support site somewhere in the dental arch than the mucosa. The former is often supported by a complex native tooth unit, consisting of enamel (or artificial crown), dentin and the PDL. Their different material properties contribute to the difference in denture displacement [109,130,216,217]. The displacement of the contact surface generally increases from the supporting tooth unit towards the distal extension (often called free-end-saddle) [218], resulting in stress concentrations in the underlying mucosa [219]. It should also be noted that the oral mucosa responds differently to loads than the PDL in a dynamic manner, as the mucosa is much easier to displace than the PDL and takes longer to recover for the same load [66,109].

In an implant-retained overdenture, the metallic implants provide even more rigid support [108], and enlarge the displacement difference at the distal ends of the denture with more severe stress concentrations, known as the cantilever effects [187,188,220,221]. Across the residual ridge, the mucosa morphology and thickness can vary significantly [39–41,45,214], and the heterogeneous bone with different qualities underneath [222–224] further contributes to the varying mechanical responses. The difference of tissue displaceability is also likely to trigger denture instability [225,226].

The tissue displaceability difference does not only cause stress-induced pain, discomfort and bone resorption [3,24–26], but also affects the long-term health of the remaining teeth and other surrounding soft tissues [131,176,227]. Several impression techniques [228–231] have been developed to minimize the effects of displaceability differences in clinical practice. Various partial denture rests (supports) have been developed and compared to reduce potential stress concentrations [75,86,113]. Shortening the denture arms [73,232] or adding a stiffer metal frame or wires [233] were suggested to reduce the cantilever effect. In the implant-retained cases, the number, location, and type of implants [82,87,108,234] have been analysed for their effects on the interaction with underlying tissues. Through all these clinical and numerical studies, understanding of the displaceability and material behaviour will contribute to enhancement of more successful treatment outcomes.

### 4.4. Residual ridge resorption

The residual ridge provides essential support to different kinds of dentures, and the bone quality is critical to the stability and functionality of a denture [3,50,235–237]. On the other hand, bone is a dynamic tissue that continuously undergoes adaptation to form a structurally elegant and efficient architecture for withstanding change of functional loads [238,239]. This adaptive process involves bone formation (apposition) and removal (resorption), which has the capability of evolving in relation to the change of habitual loading environment [207,224].

As indicated in figure 5*a*, introducing dental prostheses is likely to alter the biomechanical state in the oral structures with respect to both stimulus transfer and distribution [8,240–242]. It is believed that the alveolar bone begins to atrophy following teeth extraction or with edentulous ageing, owing to lack of stimulus to maintain the local bone quality [3,222,223,237,243–245]. However, the stimulus induced by the denture basal surface may not necessarily positively stimulate bone growth, in contrast, it may cause residual ridge resorption [3,4,235,237,243] (figure 5*d*). The established remodelling algorithms for long bones, such as Wolff's rule, are arguably inappropriate for explaining this denture-induced bone resorption [246].

From clinical observations, the residual ridge around implants often shows, to various extents, positive gains in mass density, or at least preservation of mass, [246,247]; and similar trends were present in numerical studies [86,108,187]. On the other hand, the load-borne mucosal regions often suffer from bone loss, including the posterior arms of implant-retained overdentures and the basal areas of partial or complete dentures [25,89,220,248–251], even though the stresses induced in the mucosa are much lower than those around the implants [10,13,50]. These existing studies imply that, with the soft tissue involved, residual ridge remodelling is not just the consequence of mechanical stimuli, but also affected by the physiological conditions in the surrounding tissues of mucosa, such as nutrient supply and metabolite removal to the supporting mandibular bone [15].

Clinical investigations have been exploring the aetiological pathway of denture-induced residual ridge resorption [8–12]. As pointed out in the previous sections of this review, the hydrostatic pressure in the mucosa plays a critical role, especially in the ageing population. An inappropriately designed dental prosthesis may cause further clinical complications rather than solving the initial problem of restoring masticatory function if the relevant biomechanics are not taken into account properly. In the literature, mucosal responses have gradually begun to attract considerable attention to help understand and analyse potential signs of residual ridge resorption [72,89,145,220]. However, unlike Wolff's Law, there is a lack of systematic studies on soft tissue-driven remodelling rules to guide relevant clinical activities to date.

## 5. Conclusion

With the rapid developments in molecular and cellular biology, further information has been gradually revealed as to the physiological reactions of the oral mucosa to occlusal loading, including the histological changes and biochemical reactions. Such knowledge assists with comprehending the biomechanical responses of the mucosa and provides valuable insights into the numerical modelling of these responses from clinical observations. The limitations of these biomechanical models should certainly be recognized. Proper application of these biomechanics models does not just assist with dental prosthetic design, but also enables estimation and prediction of successful treatment outcomes. Furthermore, these models can in turn contribute towards the discovery of the physiological factors associated with the biomechanical responses to advance our understanding in clinical and biological research.

This paper has reviewed four aspects of the biomechanical responses of the oral mucosa, namely the static, dynamic, volumetric and interactive responses. The first aspect, as interpreted by the assumption of linear and/or nonlinear elasticity, has been more extensively explored than the other three, and this nonlinear FEA has enabled a better match with the realistic responses of soft tissue. The dynamic response is interpreted by the viscosity component, often with assumption of homogeneity of the mucosa tissue. The heterogeneity of the mucosa has not been extensively explored as yet, which from a biomechanical perspective results in interstitial fluid activity and the associated dynamic response, thereby linking microscopic biomechanics to its physiology. The presented in-depth studies on the apparent Poisson's ratio effect and contact interaction between mucosa and dental prosthetic devices remains preliminary, and their relationship to either the mucosal anatomy or physiology remains to be clarified. Future experimental research would be appreciated in all these areas to expand the existing knowledge of mucosal biomechanics and assist clinical treatment and surgical planning for long-term success.
